# The Effects of Language Background and Parental Education on Measures of Cognitive Ability: An Analysis of the WPPSI-IV Cognitive Profiles of Monolingual, Simultaneous Bilingual, and Sequential Bilingual German Children Aged 4 to 7 Years

**DOI:** 10.3390/children11060631

**Published:** 2024-05-24

**Authors:** Franziska Walter, Monika Daseking, Franz Pauls

**Affiliations:** 1 Department of Medicine, Medical School Hamburg, 20457 Hamburg, Germany; 2 Department of Developmental and Educational Psychology, Helmut-Schmidt-University/University of the Federal Armed Forces, 22043 Hamburg, Germany; m.daseking@hsu-hh.de; 3 Department of Clinical Psychology and Psychotherapy, Helmut-Schmidt-University/University of the Federal Armed Forces, 22043 Hamburg, Germany; paulsf@hsu-hh.de

**Keywords:** German language, monolinguals, simultaneous bilinguals, sequential bilinguals, WPPSI-IV, cognitive profiles

## Abstract

Background: The present study investigated the possible effects of language background (monolinguals, simultaneous bilinguals, and sequential bilinguals) and parental education (no/low, medium, high, and highest parental education) on measures of cognitive ability provided by the Wechsler Primary and Preschool Scale—Fourth Edition (WPPSI-IV). Methods: Statistical analyses were based on a sample of 290 children (130 females, 160 males). Three multivariate variance analyses were conducted to identify possible effects. In cases of statistically significant main effects, post hoc analyses were additionally performed to identify group differences. Results: The results indicated that simultaneous bilinguals performed more similarly to monolinguals than sequential bilinguals. On average, sequential bilinguals achieved significantly lower scores on the *Verbal Comprehension Index* (VCI), the *Vocabulary Acquisition Index* (VAI), and the associated subtests than monolinguals and simultaneous bilinguals. Significantly lower average scores on VAI and the associated subtests were found for simultaneous bilinguals compared to monolinguals. Children with parents having no, a lower, or a medium educational level achieved significantly lower scores on VCI, VAI, and the FSIQ than children with parents having a high or highest educational level on average. Conclusions: The present findings suggest that the WPPSI-IV represents a suitable and reliable test battery for the assessment of cognitive skills in children with different language backgrounds and parental educational levels.

## 1. Introduction

In recent years, one of the most highly debated issues in research on bilingualism is whether bilingualism might have a beneficial or detrimental effect on the development of cognitive abilities [1]. To this very day, however, research focusing on this issue is characterized by rather inconsistent results, especially for children at young ages. Many of those inconsistencies across studies in this field may yield from the application of different methodological approaches, variations in sample selection, the use of different cognitive tests as well as the use of heterogeneous definitions of bilingualism, and the investigation of a variety of different cognitive abilities.

### 1.1. Bilingualism

In general, language users can be grouped into monolinguals, bilinguals, and multilinguals. Based on the prefixes of those terms, it can already be concluded that monolingual individuals acquire one language, bilingual ones acquire two languages, and multilingual individuals acquire more than two languages during language development. Bilinguals can be further divided into two groups, namely, those children who learn two languages simultaneously or those who learn two languages sequentially [2]. In particular, a simultaneous bilingual child can be described as an individual who acquires two languages at the same time and whose development of both languages starts in early childhood. By contrast, a sequential bilingual child can be described as an individual who learns the first language at home and is exposed to a second language later, for example, upon admittance to kindergarten or school [3].

Regarding the appropriate classification of bilinguals as either simultaneous or sequential language learners, previous studies have discussed and used age cut-offs for defining these two categories of bilingualism. For instance, McLaughlin [4] set the minimum cut-off point for the categorization into simultaneous and sequential bilinguals at age three due to the assumption that children who acquire a language before the age of three should have a considerable advantage in the according language acquisition. Others argued that the minimum cut-off point of age three may be too late for analyzing meaningful differences between the two categories. This is due to the fact that developmental differences may likely be suggested even between children who acquire two languages since birth and those who begin to learn one language between ages two and three [5,6]. Based on this, DeHouwer [7] proposed a more restricted categorization, suggesting that simultaneous bilinguals are those who were exposed to both languages from birth. A little less restrictive, Paradis, Genesee, and Crago [8] defined simultaneous bilinguals as those who learn both languages between birth and the age of one year.

### 1.2. Bilingualism and Verbal Abilities

Language acquisition is a complex process and its critical phase lies in the early lifespan of childhood [9]. Bilingual children often show reduced language abilities at the end of preschool [10,11] and regularly exhibit delayed language development as well as less pronounced language comprehension and a smaller receptive as well as expressive vocabulary [12]. On average, bilinguals show significantly smaller and more context-specific vocabularies in both languages than monolinguals. If a bilingual child speaks German at school only, for example, context-specific words associated with school, such as academic activities, objects, and subject matter, may only be known in the context-specific language (e.g., German). In this case, bilinguals show better performances in tests of expressive and receptive vocabulary in their language of instruction rather than their mother tongue because they have not built up a context-specific vocabulary in their native language [13].

In general, several studies postulate that simultaneous bilinguals perform more similarly to monolinguals than sequential bilinguals on linguistic measures, except when sequential bilinguals learn their second language in early childhood [14]. The language acquisition of children who grow up speaking two or more languages from birth is, therefore, very similar to that of monolinguals. Although bilingual children have two separate linguistic systems, those systems are closely linked [15,16,17,18].

Previous studies have already demonstrated that bilingual children often tend to score lower on receptive vocabulary tests than monolingual children of the same age in one or both languages [19,20,21]. Even though a relatively smaller vocabulary has often been found for bilingual children when comparing both languages separately with the vocabulary of monolinguals, bilinguals are suggested to not suffer from a substantial disadvantage in their language development [22,23].

### 1.3. Bilingualism and Non-Verbal Cognitive Abilities

Regarding non-verbal cognitive abilities, numerous studies have already investigated a variety of different performances of bilinguals on tasks requiring executive functioning, memory, and spatial reasoning. Even though research findings are somewhat mixed so far, it can be assumed that bilinguals may have an advantage over monolinguals, especially in solving non-verbal tasks requiring high levels of cognitive monitoring. In contrast, there are no findings that postulate that bilinguals have an advantage in tasks measuring different executive functions that require lower levels of cognitive monitoring, such as in tasks of impulse control [24,25].

Another cognitive domain of interest in research on bilingualism and non-verbal cognitive abilities is working memory. Given that research on visuospatial working memory and bilingualism has delivered consistent results [26] but the total number of studies is rather small so far, current findings on that topic still remain to be reproduced before any final conclusions can be drawn. For instance, Garcia and colleagues [27] found that bilingual children outperformed monolingual children on tasks measuring visuospatial working memory tasks, while there was no significant group difference on tasks measuring verbal working memory. The effects of bilingualism on verbal working memory even remained after controlling for demographic variables such as age, sex, socioeconomic status, and IQ. Furthermore, the authors investigated possible differences in working memory between monolingual and bilingual preschoolers with disruptive behavior disorders and concluded that bilingualism might likely be a protective factor for preschoolers with disruptive behavior disorders. In general, there are more comprehensive studies available on working memory capacity for verbal stimuli than for visual or visuospatial stimuli, but the findings on how bilingualism may affect verbal working memory are quite inconsistent. While some studies have reported an advantage for bilingual children when performing a task of working memory involving verbal stimuli [24], others have failed to demonstrate any difference between monolingual and bilingual children regarding their performances on similar tasks [28,29].

### 1.4. Bilingualism and Intelligence Testing

Besides studies suggesting an advantage or disadvantage for bilinguals in several cognitive domains such as executive functions, working memory, spatial reasoning, or verbal ability [26], there is only limited research focusing on the role of bilingualism on intelligence measures. One of the most frequently used and most accepted instruments for assessing intellectual abilities in young children [30] is the Wechsler Primary and Preschool Scale of Intelligence—Fourth Edition (WPPSI-IV; [31]). While studies investigating differences in intellectual abilities between monolingual and bilingual children have used comprehensive intelligence test batteries, such as the Wechsler Intelligence Scale for children such as WISC-IV [32], such analyses were mostly conducted based on the data of children older than 6 years.

Bialystok and Majumder [33] analyzed differences between the performances of monolingual and bilingual 7- to 9-year-old children on the WISC-IV subtest *Block Design* and found that bilinguals achieved higher scores than monolinguals. In another study focusing on four WISC-IV subtests, (*Block Design*, *Digit Span*, *Vocabulary*, and *Arithmetic*) Lauchlan, Parisi, and Fadda [34] found an advantage for bilinguals in the subtests *Block Design* and *Vocabulary*, but no significant mean score differences for the subtests *Digit Span* and *Arithmetic* when compared to monolinguals. Karlsson et al. [35] examined the effect of language background on ten WISC-IV subtests in children aged 7 and 10 to 11 and found that monolingual children of the younger age group on average achieved a significantly higher score on the subtest *Symbol Search* than bilinguals. When analyzing verbal visual attention measured with the WISC-IV subtest *Cancellation* of WISC-IV, Calvo and Bialystok [36] demonstrated that there was no difference between the performances of monolingual and bilingual children.

Schweizer et al. [37] investigated differences among monolinguals, simultaneous bilinguals, and sequential bilinguals regarding general intelligence and seven factors with two subtests each provided by the Intelligence and Development Scales—2 (IDS—2, [38]). Based on their sample, including children and adolescents aged 5 to 20, the results indicated lower scores for sequential bilinguals on general intelligence, verbal reasoning, and verbal long-term memory when compared to monolinguals and simultaneous bilinguals. However, there were almost no significant differences found between the performances of simultaneous bilinguals and monolinguals on any IDS-2 measure under examination.

Given the lack of research on this topic, the present study sets out to investigate the potential differences between the performances of monolingual and bilingual children on intellectual measures provided by the German WPPSI-IV [39].

### 1.5. Parental Education and Cognitive Abilities

As described above, studies on the role of bilingualism in cognitive abilities have suggested effects of bilingualism vary in their direction depending on whether verbal or non-verbal abilities were investigated [40]. In contrast to these findings regarding bilingualism, studies on sociodemographic factors found relatively strong and consistent effects of parental education and socioeconomic status on cognitive abilities [10].

Parental education is considered one of the best predictors of intelligence in children as well as their school achievement [41]. Recent studies have demonstrated that a higher parental educational level is related to higher levels of intelligence in children and better school performance due to other family-related factors such as social and material resources [42]. Generally, the heritability of intelligence is high; the relationship between parents’ intelligence and children’s intelligence already shows in early childhood, then increases in adolescence, and displays its highest relationship in adulthood [43].

Eilertsen and colleagues [44] investigated the relationship between socioeconomic status (SES; including parental education) and cognitive functioning measured with the Wechsler Intelligence Scale for Children–Third Edition (WISC-III, [45]) in a sample of Norwegian children aged 8 to 12 years. The results indicated a strong association between SES and the Verbal Comprehension Index, in which maternal education was the only significant predictor of SES. Lower significant relationships were also found between SES and *Full-Scale* IQ as well as SES and *Working Memory* IQ.

### 1.6. Bilingualism and Parental Education

It may be assumed that performances on cognitive tasks might also be affected by an interaction between bilingualism and socioeconomic status so that the main effects of one depend on certain levels of the other [36]. It is, thus, possible that only those bilingual children featuring a specific level of parental education, such as the highest educational level, appear to have an advantage in tasks measuring working memory capacity while bilinguals featuring other levels of parental education do not [36].

Since bilingualism and parental education both are suggested to be strongly correlated or interdependent, thus sharing a great portion of common variance, it is challenging to examine the possible effects of both on cognitive abilities separately. Besides examining possible interaction effects of bilingualism and parental education on cognitive abilities, however, it is also deemed necessary to distinguish between possible effects of each of those factors while controlling for the other.

Daseking et al. [46] investigated the role of migration background on intellectual abilities in a sample of German children and adolescents aged 6 to 16 using the *German Wechsler Intelligence Scale for Children—Fifth Edition* (WISC-V, [47]). The sample was divided into two different groups: a group of younger children attending primary school and a second group of older children or adolescents attending secondary school. In the group of younger children, the possible effects of parental education and migration background were analyzed on primary and ancillary index scores as well as on the *Full-Scale IQ* (FSIQ). In the group of older children and adolescents, the attended school type was additionally included in the analyses as an additional potentially contributing factor for intellectual measures. The results of the study indicated that differences in the corresponding WISC-V index scores between children and adolescents with and without migration background could be fully explained either by parental education (in the group of younger children) or by the attended school type (in the group of older children and adolescents). Depending on their migration background, however, children and adolescents with a migration background were also found to attend school types associated with lower levels of education more frequently than those without a migration background.

### 1.7. Research Questions

Since there is only a small body of research suggesting quite diverse results on the role of language background on cognitive profiles, the need to provide more extensive investigations on this topic is evident. For this purpose, using measures of intellectual abilities that are provided by the widely used Wechsler Scales is required to reliably reflect those intellectual abilities to be examined, thus enabling the estimation of valid cognitive profiles. In particular, studies specifically focusing on the differences between young monolingual, simultaneous bilingual, and sequential bilingual children regarding their intellectual abilities are still missing despite their relevant implications for clinical practice. Especially in borderline cases, for instance, clinical decisions based on measures of intellectual abilities might likely be flawed due to the failure to consider the possible impact of language background on test performances.

There has been little research to date, and the results of this research on the role of language background in cognitive profiles vary considerably. For a more comprehensive study on this topic, the intellectual abilities of young children should be measured using the widely used Wechsler scales, which reliably reflect the intellectual abilities to be analyzed and, thus, enable the creation of valid cognitive profiles. Particularly for borderline performance, clinical decisions based on measures of intellectual ability may be flawed because the possible influence of language background on test performance is not adequately taken into account. As a result, children may not receive the necessary support or therapeutic assistance.

In everyday life, not only for parents but also for educational professionals, the question repeatedly arises as to the order in which the mother tongue (first language) and other languages (here in particular the national language or language of education) are acquired most successfully and how children and families can be supported in this process, especially that the mastery of the language of education can be regarded as essential for educational success.

Even though intelligence tests such as the Wechsler Scales were not primarily developed to assess intellectual abilities associated with language background, the 15 WPPSI-IV subtests, the primary and ancillary indices, and the FSIQ can still provide suitable measures for the comparison between young monolingual and bilingual children. In that regard, the current study aims to clarify whether language background (monolingual, simultaneous bilingual, and sequential bilingual) and parental education (no/low educational level, medium educational level, high educational level, and the highest educational level) can be suggested to have a remarkable impact on the WPPSI-IV test performances of children aged 4 to 7 as reflected by their scores on the primary and ancillary index, the 15 subtests, and the FSIQ.

On the basis of these considerations, the present study aims to address the following research questions:

1. Do scores on the WPPSI-IV primary indices and Full-Scale IQ significantly differ between children aged 4 to 7 years depending on their language background (monolingual, simultaneous bilingual, sequential bilingual) and parental education (no/low educational level, medium educational level, high educational level, and the highest educational level)? Is there an interaction effect between language background and parental education on the performances on the WPPSI-IV primary indices in children aged 4 to 7 years?
2. Do scores on the WPPSI-IV ancillary indices significantly differ between children aged 4 to 7 years depending on their language background (monolingual, simultaneous bilingual, sequential bilingual) and parental education (no/low educational level, medium educational level, high educational level, and the highest educational level)? Is there an interaction effect between language background and parental education on the performances on the WPPSI-IV ancillary indices in children aged 4 to 7 years?
3. Do performances on the WPPSI-IV subtests significantly differ between children aged 4 to 7 years depending on their language background (monolingual, simultaneous bilingual, sequential bilingual) and parental education (no/low educational level, medium educational level, high educational level, and the highest educational level)? Is there an interaction effect between language background and parental education on the performances on the WPPSI-IV subtests in children aged 4 to 7 years?

## 2. Materials and Methods

### 2.1. Sample

Subsequent analyses were based on the data of a sample of *N* = 290 children (130 females, 160 males), which was selected from the extended dataset of the German WPPSI-IV standardization sample (see Table 1 for an overview). One parent per child completed a questionnaire assessing sociodemographic variables such as sex, age, parental education, and language background. The data from monolinguals were matched to the data from the group of simultaneous bilinguals and sequential bilinguals for age, sex, and parental education. Children were recruited from different kindergartens and elementary schools in Bremen, Lower Saxony, Saxony, Baden-Wuerttemberg, North Rhine-Westphalia, and Hamburg, Germany. Prior to data collection and testing, parents and (kindergarten) teachers were informed about the main goals and procedures of the study as well as about data processing and protection. Parents were then required to give their approval by signing a written informed consent form.

The exclusion criteria for the sample were the following: the child does not speak/understand German; the child participated in another intelligence testing during the last six months; the child has significant limitations of the upper and lower extremities; the child takes medication that may affect test performances (e.g., anticonvulsants); or the child has a diagnosed physical, neurological, or mental disorder that could significantly influence test performances (e.g., stroke, brain tumor, epilepsy).

Since previous research has suggested that performances on cognitive tasks and the language use of multilingual children are not comparable to those of bilingual and monolingual children due to qualitative differences [48], multilingual children were excluded from the data analyses of the present study. The less restrictive definition for simultaneous bilinguals proposed by Paradis, Genesee, and Crago [8] was used in the present study: children who learn both languages between birth and the age of one year are classified as simultaneous bilinguals, as this classification reflects a compromise between the restrictive and less restrictive definition. During testing, only German was spoken to the participating children and all tasks were presented in German.

### 2.2. Measures

#### 2.2.1. Language Background and Parental Education

Language background and parental education were measured using specific questions within a sociodemographic questionnaire that was completed by one parent (see Table 1 for an overview of the questions addressing language background). Language background is defined as the number of acquired languages and the way they were acquired during language development. Parental education is defined as the highest level of education achieved by either one parent or both: 1 = low educational level, 2 = medium educational level, 3 = high educational level, 4 = highest educational level.

#### 2.2.2. Wechsler Preschool and Primary Scale—Fourth Edition (WPPSI-IV)

The German Wechsler Preschool and Primary Scale of Intelligence—Fourth Edition (WPPSI-IV; [39]) provides different measures for evaluating the intellectual abilities of children aged 2 years and 6 months to 7 years and 7 months. The WPPSI-IV provides two age group versions: one for children aged 2 years and 6 months to 3 years and 11 months (2:6–3:11) and the other one for children aged 4 years to 7 years and 7 months (4:0–7:7).

The WPPSI version for ages 4:0–7:7 includes a total of 15 subtests, each measuring specific and relatively independent intellectual abilities: *Information* (IN), *Receptive Vocabulary* (RV), *Picture Naming* (PN), *Block Design* (BD), *Object Assembly* (OA), *Picture Memory* (PM), *Zoo Locations* (ZL), *Similarities* (SI), *Vocabulary* (VO), *Comprehension* (CO), *Matrix Reasoning* (MR), *Picture Concepts* (PC), *Bug Search* (BS), *Cancellation* (CA), and *Animal Coding* (AC). Each subtest produces a raw score, which is transformed into a scaled score that can range from 1 to 19 (standard scores; *M* = 10, *SD* = 3), with scores between 7 and 12 usually considered average. The *Full-Scale IQ* (FSIQ), representing general intelligence, and 5 primary index scores for the *Verbal Comprehension Index* (VCI), *Visual Spatial Index* (VSI), *Fluid Reasoning Index* (FRI), *Working Memory Index* (WMI), and *Processing Speed Index* (PSI), representing five broad cognitive abilities, can be derived from the subtest scaled scores. In addition, ancillary index scores can also be calculated for the *Vocabulary Acquisition Index* (VAI), *Nonverbal Index* (NVI), *General Ability Index* (GAI), and *Cognitive Proficiency Index* (CPI). The FSIQ and all index scores can range from 40 to 160 (standard scores; *M* = 100, *SD* = 15), with scores from 85 to 114 considered average. The five primary index scores include those factor-based composite scores representing the main cognitive domains that are typically obtained for a comprehensive evaluation of cognitive functioning. The four ancillary index scores are theory-driven and may be used to provide additional or supporting information regarding a child’s WPPSI–IV performances.

For the German adaptation of the WPPSI-IV, the primary indices VCI, VSI, FRI, and WMI and the ancillary indices VAI, NVI, GAI, and CPI have indicated excellent internal consistency coefficients across all ages, with Cronbach’s alpha ranging from 0.87 to 0.94. The test–retest reliability coefficients for the PSI subtests, ranging from 0.72 to 0.74, turned out to be lower in value but still with sufficiently good stability.

### 2.3. Data Analysis

All analyses of the present study were performed using the IBM SPSS Statistics software (Version 29), and an alpha level of 0.05 for statistical significance was set for all statistical tests. First, the total sample of *N* = 290 children aged 4 to 7 years was scanned for outliers, deviations from multivariate normality (e.g., skewness and kurtosis), and homogeneity of the covariance matrices. In order to answer the research questions of the present study, three multivariate analyses of variance (MANOVA) were performed to evaluate the statistical significance of multivariate effects using Wilk’s λ as well as univariate main and interaction effects using *F* statistics. A first multivariate analysis of variance (MANOVA 1) was conducted to test for the hypothesized main and interaction effects of language background and parental education on the five WPPSI-IV primary indices and the FSIQ. Possible main and interaction effects of language background and parental education on two WPPSI-IV ancillary indices *Vocabulary Acquisition Index* (VAI) and *Nonverbal Index* (NVI) were then analyzed by performing a second multivariate analysis of variance (MANOVA 2). A third multivariate analysis of variance (MANOVA 3) was finally conducted to test for the hypothesized main and interaction effects of language background and parental education on the 15 WPPSI-IV subtests. Partial eta squared (η^2^_par_) was used to evaluate the effect sizes of statistically significant main and interaction effects, with η^2^_par_ ≥ 0.01 indicating a small effect, η^2^_par_ ≥ 0.06 indicating a moderate effect, and η^2^_par_ ≥ 0.14 indicating a large effect. In cases of statistically significant main effects, post hoc analyses were additionally performed by comparing means of specific factor levels to identify group differences that may explain the corresponding main effect. The Bonferroni method was used to adjust for multiple post hoc comparisons to control for the overall probability of a Type I error for multiple hypothesis tests.

## 3. Results

### 3.1. Descriptive Statistics

#### 3.1.1. Sample

Sociodemographic characteristics for the groups of monolinguals, simultaneous bilinguals, and sequential bilinguals are depicted in Table 2.

The quality of data matching was checked using chi-square tests of homogeneity and the Kruskal–Wallis test statistics for nonparametric data. Both test statistics indicated that there were no differences between the groups of monolinguals, simultaneous bilinguals, and sequential bilinguals regarding their distributions of parental education (χ^2^ (6, *N* = 290) = 0.586, *p* = 0.997, age (H (2, *N* = 290) = 0.377, *p* = 0.828) and sex (χ^2^ (2, *N* = 290) = 0.030, *p* = 0.985).

#### 3.1.2. Test Performances

As indicated by Box’ M test statistics and further investigations of box plots and skewness and kurtosis values, no extreme outliers and no substantial deviations from the multivariate normality or homogeneity of the covariance matrices could be found for the data of monolinguals, simultaneous bilinguals, and sequential bilinguals.

Table 3 presents descriptive statistics, suggested means, and standard deviations for the scaled scores on all 15 WPPSI-IV, the five primary index scores, two ancillary index scores, and FSIQ for the three groups of language background, as well as the total sample.

### 3.2. Multivariate Analysis of Variance on the WPPSI-IV Primary Index Scores and the FSIQ

The results of the multivariate analysis of variance 1 (MANOVA 1), including the main and interaction effects of language background and parental education on the five WPPSI-IV primary index scores and the FSIQ are presented in Table 4. MANOVA 1 yielded significant multivariate effects of language background (Wilks’s λ = 0.700, *F*(12, 548) = 2.592, *p* = 0.013, η^2^_par_ = 0.045) and parental education (Wilks’s λ = 0.912, *F*(8, 842) = 1.952, *p* = 0.010, η^2^_par_ = 0.041).

**Figure 1:**
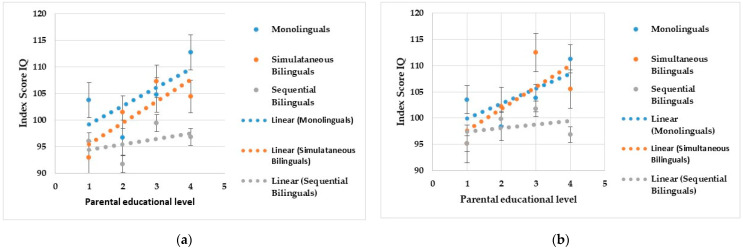
Means for (**a**) the VCI scores and (**b**) the FSIQ scores across the groups of parental education and language background. *Note.* Language background is defined as the number of acquired languages and the way they were acquired during language development (1 = simultaneous bilingual, 2 = sequential bilingual, 3 = monolingual). Parental education is defined as the highest level of education achieved by either one parent or both (1 = no to low educational level, 2 = medium educational level, 3 = high educational level, 4 = highest educational level). VCI = Verbal Comprehension Index, FSIQ = Full Scale IQ.

Univariate analyses indicated small but significant effects of language background on VCI (*F*(2, 278) = 8.535, *p* < 0.001, η^2^_par_ = 0.058) and the FSIQ (*F*(2, 278) = 4.499, *p* = 0.012, η^2^_par_ = 0.031). Furthermore, significant univariate effects of parental education were found on VCI (*F*(3, 278) = 5.817, *p* < 0.001, η^2^_par_ = 0.059) as well as on the FSIQ (*F*(3, 278) = 4.260, *p* = 0.006, η^2^_par_ = 0.044). Even though no multivariate interaction effect was found, a univariate effect of the interaction between language background and parental education turned out to be significant for VCI (*F*(6, 278) = 2.290, *p* = 0.036, η^2^_par_ = 0.047), WMI (*F*(2, 278) = 2.507, *p* = 0.022, η^2^_par_ = 0.051), and for the FSIQ (*F*(2, 278) = 2.851, *p* = 0.010, η^2^_par_ = 0.058). Figure 1a) illustrates the interaction between language background and parental education on VCI and the interaction effect for the FSIQ is illustrated in Figure 1b).

The results of the post hoc analyses for the main effects in MANOVA 1 are presented in Table 5. On average, sequential bilinguals showed significantly lower VCI scores than simultaneous bilinguals (Δ*M* = 6.06; *SE* = 2.355; *p* = 0.032) and monolinguals (Δ*M* = −9.15; *SE* = 1.996; *p* < 0.001). Moreover, children with a sequential bilingual language background achieved significantly lower FSIQ scores on average than monolingual children (Δ*M* = −6.38; *SE* = 1.975; *p* = 0.004).

Furthermore, post hoc analyses also indicated that, on average, children with parents having no or a low educational level achieved significantly lower VCI scores than children with parents having the highest educational level (Δ*M* = −7.49; *SE* = 2.274; *p* = 0.007). Significantly lower VCI scores were also found for children with parents having a medium educational level compared to children with parents having a high educational level (Δ*M* = −7.61; *SE* = 2.540; *p* = 0.018) and the highest educational level (Δ*M* = −10.63; *SE* = 2.161; *p* < 0.001). In addition, children with parents having the highest educational level were found to achieve higher FSIQ scores on average than children with parents having no or a low educational level (Δ*M* = −6.92; *SE* = 2.250; *p* = 0.014) and a medium educational level (Δ*M* = −6.66; *SE* = 2.138; *p* < 0.012).

### 3.3. Multivariate Analysis of Variance on the WPPSI-IV Ancillary Index Scores

Table 6 presents a summary of the multivariate analysis of variance 2 (MANOVA 2) with the main and interaction effects of language background and parental education on two WPPSI-IV ancillary index scores. The results indicated significant multivariate effects on two WPPSI-IV ancillary index scores of language background (Wilks’s *λ* = 0.804, *F*(4, 554) = 15.959, *p* < 0.001, η^2^_par_ = 0.103), parental education (Wilks’s *λ* = 0.936, *F*(6, 554) = 3.112, *p* = 0.005, η^2^_par_ = 0.033), and the language background × parental education interaction (Wilks’s *λ* = 0.923, *F*(12, 554) = 1.883, *p* = 0.034, η^2^_par_ = 0.039).

Furthermore, the analysis yielded significant univariate effects of language background on VAI (*F*(2, 278) = 29.473, *p* < 0.001, η^2^_par_ = 0.175) and univariate effects of parental education on VAI (*F*(3, 278) = 4.432, *p* = 0.005, η^2^_par_ = 0.046). The language background × parental education interaction seemed to be significant for NVI (*F*(6, 278) = 2.702, *p* = 0.014, η^2^_par_ = 0.055).

A summary of the results of the post hoc analyses for the main effects of language background and parental education on two WPPSI-IV ancillary indices (MANOVA 2) are presented in Table 7. The results showed that on average, sequential bilinguals demonstrated significantly lower performances than simultaneous bilinguals (Δ*M* = 8.04; *SE* = 2.167; *p* < 0.001) and monolinguals (Δ*M* = −15.15; *SE* = 1.836; *p* < 0.001) on VAI. But also, children growing up simultaneously bilingual indicated significantly lower scores on VAI than monolingual children (Δ*M* = −7.11; *SE* = 1.914; *p* < 0.001).

Table 7 also presented the results about children with parents having the highest educational level, the post hoc analysis indicated higher performances on average for these children on VAI than children with parents having the lowest educational level (Δ*M* = −6.50; *SE* = 2.093; *p* = 0.013), as well as children with parents having a medium educational level (Δ*M* = −7.71; *SE* = 1.988; *p* < 0.001).

### 3.4. Multivariate Analysis of Variance on the WPPSI-IV Subtest Scaled Scores

A summary of the multivariate analysis of variance 3 (MANOVA 3) with the main and interaction effects of language background and parental education on the 15 WPPSI-IV subtest scaled scores is presented in Table 8. The analysis demonstrated significant multivariate effects on the 15 WPPSI-IV subtests scaled scores of language background (Wilks’s *λ* = 0.724, *F*(30, 528) = 3.083, *p* < 0.001, η^2^_par_ = 0.149) and parental education (Wilks’s *λ* = 0.737, *F*(45, 785) = 1.885, *p* < 0.001, η^2^_par_ = 0.097).

All results of the univariate analyses including the main and interaction effects of language background and parental education on the 15 WPPSI-IV subtest scaled scores (MANOVA 3) are presented in Table 8.

An interaction effect of language background × parental education appeared to be significant for the following subtests: SI (*F*(6, 278) = 2.154, *p* = 0.048, η^2^_par_ = 0.044) and VC (*F*(6, 278) = 2.234, *p* = 0.040, η^2^_par_ = 0.046).

The significant post hoc analyses are too numerous to include in this section. A summary of the results of post hoc analyses for the main effects of language background and parental education on the WPPSI-IV subtests scaled scores (MANOVA 3) are presented in Table 9.

## 4. Discussion

The major aim of the present study was to investigate whether performances on the five WPPSI-IV primary indices and the FSIQ, the two ancillary indices (*Vocabulary Acquisition Index*, *Nonverbal Index*), and the 15 WPPSI-IV subtests differ between children aged 4 to 7 years depending on their language background (monolingual, simultaneous bilingual, and sequential bilingual) and parental education (no/low educational level, medium educational level, high educational level, and the highest educational level). Furthermore, a second aim of the present study was to examine if there is an interaction effect between language background and parental education on the performances on the same WPPSI-IV measures in children aged 4 to 7 years.

As indicated by the results of the multivariate analyses of variance, the group of sequential bilinguals showed lower scores on primary and ancillary indices, as well as on subtests measuring verbal abilities compared to monolinguals and simultaneous bilinguals. On average, monolinguals appeared to outperform sequential bilinguals in the WPPSI-IV indices *Verbal Comprehension Index* (VCI), *Vocabulary Acquisition Index* (VAI), *the Full-Scale IQ* (FSIQ), and the WPPSI-IV subtests *Information* (IN), *Similarities* (SI), *Vocabulary* (VO), *Comprehension* (CO), *Receptive Vocabulary* (RV), *Picture Naming* (PN), and *Object Assembly* (OA). Simultaneous bilinguals were found to outperform sequential bilinguals in the WPPSI-IV indices VCI and VAI as well as in the WPPSI-IV subtests IN, VO, CO, RV, and PN. However, simultaneous bilinguals achieved significantly lower scores than monolinguals only on VAI, RV, and PN. It should be noted that the subtests RV and PN are both used to compute VAI.

These findings are for the most part in line with the current research [14,15,16,17,18,37], postulating that the average levels of performance of simultaneous bilinguals on verbal tasks are more similar to monolinguals than to sequential bilinguals. For this reason, it may be suggested that language acquisition in children who grow up speaking two languages from birth may be very similar to that of monolinguals, as the two separate linguistic systems of simultaneous bilinguals are closely linked.

In addition to verbal abilities, crystallized intelligence, long-term memory, the ability to retain and retrieve knowledge from the environment, verbal perception, comprehension, and expression are required for solving tasks that are related to VCI and the associated subtests IN, SI, VC, and CO. However, if applying a specific vocabulary fails, those tasks can also be completed by paraphrasing the vocabulary. While simultaneous bilinguals may solve such tasks just as well as monolinguals, sequential bilinguals might lack the vocabulary to solve the tasks for VCI and the associated subtests as well as monolingual children. Furthermore, it may be suggested that simultaneous bilinguals tend to outperform sequential bilinguals on VCI and the associated subtests because they learned German in early childhood and, therefore, have probably a more comprehensive vocabulary.

However, some studies have already indicated that bilinguals, including simultaneous bilinguals, tend to score lower on tests measuring receptive vocabulary than monolinguals [19,20,21]. By contrast, VAI and the associated subtests RV and PN measure developmental vocabulary acquisition and are primarily based on expressive and receptive vocabulary. For solving tasks that are related to VAI, RV, and PN, applying the exact vocabulary is required. Given that the vocabulary of simultaneous bilinguals is divided into two closely linked but still separate linguistic systems, simultaneous bilinguals may show lower performances on VAI and the associated subtests than monolinguals.

Furthermore, the potential effects of parental education on the children’s performances regarding the WPPSI-IV primary indices, the FSIQ, two ancillary indices, and the 15 WPPSI-IV subtests were analyzed. The present findings indicated the effects of parental education on similar index and subtest scores that were also found to be affected by language background. In particular, children with parents having a low to medium educational level were found to achieve lower scores on VCI, VAI, and the associated subtests IN, SI, VC, CO, RV, and PN than children with parents having a high or the highest educational level.

These findings are in line with the findings of Eilertsen and colleagues [44], who investigated the relationship between socioeconomic status (SES) and cognitive functioning measured with the WISC-III. As part of the SES, maternal education was found to be the only significant predictor explaining the strong association between SES and VCI. Similarly to the present findings, Eilertsen and colleagues [44] also suggested a small but significant association between the SES and the FSIQ.

Several studies have already emphasized a positive relationship between SES and productive vocabulary, which might likely be influenced by the mean length of utterances in parental speech, especially maternal speech [49]. In addition, Raviv, Kessenich, and Morrison [50] investigated possible mediators for the relationship between SES and children’s language skills and concluded that maternal sensitivity and cognitive stimulation might partially mediate the effects of SES on verbal comprehension, expressive language, and receptive verbal conceptual abilities.

In addition to the main effects of language background and parental education, the present findings also suggested a significant interaction effect of language background and parental education on VCI and the FSIQ. As depicted in Figure 1a), monolinguals and simultaneous bilinguals were found to achieve higher scores on VCI than sequential bilinguals with increasing parental educational levels. While performances on the corresponding tasks were not significantly different across all three groups of language background at the low parental educational level, monolinguals and simultaneous bilinguals both outperformed sequential bilinguals the most at the high or highest parental educational level. A similar interaction effect of language background and parental education was also found on the FSIQ (see Figure 1b). Monolinguals and simultaneous bilinguals were found to achieve higher scores on the FSIQ than sequential bilinguals with increasing parental educational levels. At the low parental educational level, monolinguals, simultaneous bilinguals, and sequential bilinguals achieved on average comparable scores on the FSIQ, whereas monolinguals and simultaneous bilinguals both outperformed sequential bilinguals the most at the high or highest parental educational level.

As Figure 1 illustrates, the interaction effects of language background and parental education on both VCI and the FSIQ were found to be due to the increasing associated scores for monolinguals and simultaneous bilinguals with increasing parental educational levels. In contrast, the level of performance in sequential bilinguals appeared to be quite similar across all parental educational levels, thus being independent of parental education.

Even though these findings indicated no substantial effect of parental education, the test performances of sequential bilinguals regarding VCI and the FSIQ seem to be in line with previous studies suggesting a strong association between maternal education and children’s productive vocabulary as well as language skills [49,50]. It should be noted that the group of sequential bilinguals includes children who learned to speak German at the earliest age of two years and at the latest when they attended kindergarten. However, this could mean that parents have not spoken German with their children by this time and have, therefore, not been able to promote or teach German to their children.

In summary, the findings of the present study clearly suggest that performances on verbal tasks as reflected by VCI, VAI, and the associated subtests may be affected by parental education and language background. Especially in the cases of children with parents having a low to medium educational level, it is, therefore, advisable to use nonverbal tasks and measures such as the *Nonverbal Index* (NVI) to provide a measure of cognitive ability less dependent on language. Given that the impact of parental education and language background could be at least partly due to limitations in the structural validity of the WPSSI-IV regarding these specific groups, future studies should investigate measurement invariance on the WPPSI-IV across different parental educational levels and different groups of language background. Moreover, future research should clarify whether those effects of language background and parental education in early childhood are also evident in development in later childhood, adolescence, and adulthood as well.

### Limitations

There are some limitations in the present study that need to be addressed and should also be considered when replicating or interpreting the present results.

As the present study is cross-sectional in nature, inferences about causality are limited by definition. While there is a theoretical and empirical basis for conducting multivariate variance analyses on cross-sectional data in the research literature, however, further analyses on longitudinal data are required to investigate more causal effects of language background and parental education. This way, longitudinal studies might then provide a more adequate insight into possible causal effects of language background and parental education on children’s cognitive development over time.

It should also be noted that the WPPSI-IV was the only test battery that was used to measure and operationalize cognitive abilities in the present study. Since using alternative and more diverse approaches could improve the reliability of measurements by reducing potential methodological biases and test-specific limitations, future research should focus on multi-methodological measurements of cognitive abilities when investigating possible effects on test performances that may be influenced by parental education and language in early childhood.

Another limitation of the present study is the heterogeneity of the sample. Although it was determined that all children participating in the study spoke German as their first or second language, other spoken languages could be assigned to a variety of different language groups. Since those language groups were not further investigated, the representativeness and comparability of the simultaneous and sequential bilinguals cannot be fully guaranteed. Accordingly, the level of development of the language that was not German was not measured or determined. Future research should, thus, clarify to what extent the present findings can be transferred to different language groups.

## 5. Conclusions

Despite the aforementioned limitations, the present study represents a further contribution to the research on the effects of language background and parental education on the WPPSI-IV test performances of monolingual, simultaneous bilingual, and sequential bilingual German children aged 4 to 7 years. First of all, simultaneous bilinguals tend to perform more similarly to monolinguals than to sequential bilinguals. Sequential bilinguals were found to be outperformed by monolinguals and simultaneous bilinguals on most verbal tasks, achieving lower scores on VCI and VAI, and on the verbal subtests IN, VC, CO, RV, and PN. Compared to monolinguals, however, simultaneous bilinguals also achieved lower scores on VAI and the associated subtests PN and RV. Moreover, the present findings also indicated that parental education may likely affect the performances on the WPPSI-IV; this is because children with parents having a lower educational level tend to achieve lower scores on VCI, VAI, the FSIQ, and the subtests IN, SI, VC, CO, RV, and PN than children with parents having a higher educational level. In general, future research should clarify whether those effects of language background and parental education might at least partially explain variations in cognitive abilities in later childhood, adolescence, and adulthood as well.

## Figures and Tables

**Table 1:** Questions for measuring language background by classifying children as monolingual, simultaneous bilingual, and sequential bilingual.

Question	Monolingual	Simultaneous Bilingual	Sequential Bilingual
Does the child only understand/speak German? (If so, the parents no longer needed to answer the subsequent questions about language background)	Yes	No	No
Does the child only understand/speak a language other than German?	-	No	No
Does the child understand/speak German and another language?	-	Yes	Yes
What other language does the child speak/understand besides German?	-	Just one other language besides German	Just one other language besides German
At what age did the child speak/understand German?	-	Birth to 1	>1
At what age did the child go to the kindergarten?	-	Within one year at the latest	Starting at age 2 at the earliest

*Note.* Language background is defined as the number of acquired languages and the way they were acquired during language development. Parental education is defined as the highest level of education achieved by either one parent or both.

**Table 2:** Sociodemographic characteristics for the groups of monolinguals, simultaneous bilinguals, and sequential bilinguals (*N* = 290).

	Monolingual (*n* = 145)	Simultaneous Bilingual (*n* = 68)	Sequential Bilingual (*n* = 77)
Age in months: *M* (*SD*)	68.05 (11.84)	67.57 (11.97)	68.32 (11.92)
	*n* (%)	*n* (%)	*n* (%)
Sex (female)	65 (44.8)	31 (45.6)	34 (44.2)
Parental education:			
1 = no to low educational level	34 (23.4)	15 (22.1)	19 (24.7)
2 = medium educational level	41 (28.3)	18 (26.5)	23 (29.9)
3 = high educational level	25 (17.2)	12 (17.6)	13 (16.9)
4 = highest educational level	45 (31.0)	23 (33.8)	22 (28.6)

*Note.* Language background is defined as the number of acquired languages and the way they were acquired during language development. Parental education is defined as the highest level of education achieved by either one parent or both.

**Table 3:** Descriptive statistics (means and standard deviations) on the five WPPSI-IV primary index scores, the FSIQ, two ancillary index scores, and 15 subtests for the total sample and language background groups.

Variables	Descriptive Statistics			
		*N*	*M*	*SD*
Verbal Comprehension Index	simultaneous bilingual	68	101.62	13.32
	sequential bilingual	77	95.56	14.11
	monolingual	145	104.70	15.92
	Total sample	290	101.55	15.31
Visual Spatial Index	simultaneous bilingual	68	104.16	16.62
	sequential bilingual	77	100.70	14.43
	monolingual	145	105.14	15.10
	Total sample	290	103.73	15.60
Fluid Reasoning Index	simultaneous bilingual	68	104.41	15.37
	sequential bilingual	77	98.98	13.01
	monolingual	145	102.92	15.86
	Total sample	290	102.23	15.13
Working Memory Index	simultaneous bilingual	68	102.54	14.36
	sequential bilingual	77	99.74	14.38
	monolingual	145	103.34	14.88
	Total sample	290	102.20	14.65
Processing Speed	simultaneous bilingual	68	100.69	12.50
	sequential bilingual	77	101.69	14.78
	monolingual	145	102.95	15.57
	Total sample	290	102.09	14.67
Full-Scale IQ	simultaneous bilingual	68	103.57	13.48
	sequential bilingual	77	98.16	14.08
	monolingual	145	104.53	15.25
	Total sample	290	102.61	14.75
Vocabulary Acquisition Index	simultaneous bilingual	68	97.32	12.61
	sequential bilingual	77	89.29	13.06
	monolingual	145	104.43	13.77
	Total sample	290	98.74	14.72
Nonverbal Index	simultaneous bilingual	68	104.66	14.99
	sequential bilingual	77	99.82	13.88
	monolingual	145	104.00	15.19
	Total sample	290	103.04	14.89
Block Design	simultaneous bilingual	68	10.91	3.37
	sequential bilingual	77	10.19	2.86
	monolingual	145	10.60	3.22
	Total sample	290	10.57	3.17
Information	simultaneous bilingual	68	10.21	2.59
	sequential bilingual	77	8.87	2.97
	monolingual	145	10.85	3.01
	Total sample	290	10.17	3.01
Matrix Reasoning	simultaneous bilingual	68	10.69	3.02
	sequential bilingual	77	9.84	2.86
	monolingual	145	10.38	3.18
	Total sample	290	10.31	3.07
Bug Search	simultaneous bilingual	68	10.40	2.79
	sequential bilingual	77	10.06	3.30
	monolingual	145	10.31	3.20
	Total sample	290	10.27	3.13
Picture Memory	simultaneous bilingual	68	10.38	3.13
	sequential bilingual	77	9.84	2.84
	monolingual	145	10.86	2.97
	Total sample	290	10.48	3.13
Similarities	simultaneous bilingual	68	10.47	3.10
	sequential bilingual	77	9.68	2.81
	monolingual	145	10.91	3.20
	Total sample	290	10.48	3.11
Picture Concepts	simultaneous bilingual	68	10.82	3.23
	sequential bilingual	77	9.82	2.67
	monolingual	145	10.62	3.06
	Total sample	290	10.46	3.02
Cancellation	simultaneous bilingual	68	9.76	2.59
	sequential bilingual	77	10.42	2.63
	monolingual	145	10.59	3.09
	Total sample	290	10.35	2.87
Zoo Locations	simultaneous bilingual	68	10.47	2.48
	sequential bilingual	77	10.04	2.80
	monolingual	145	10.25	2.98
	Total sample	290	10.24	2.82
Object Assembly	simultaneous bilingual	68	10.40	2.93
	sequential bilingual	77	9.95	2.87
	monolingual	145	11.07	2.90
	Total sample	290	10.61	2.93
Vocabulary	simultaneous bilingual	68	10.54	2.79
	sequential bilingual	77	8.86	3.01
	monolingual	145	10.77	2.62
	Total sample	290	10.21	2.88
Animal Coding	simultaneous bilingual	68	10.26	2.82
	sequential bilingual	77	10.51	3.16
	monolingual	145	10.66	3.06
	Total sample	290	10.52	3.03
Comprehension	simultaneous bilingual	68	10.38	2.78
	sequential bilingual	77	9.01	2.89
	monolingual	145	10.26	2.96
	Total sample	290	9.96	2.94
Receptive Vocabulary	simultaneous bilingual	68	9.60	2.57
	sequential bilingual	77	8.21	2.53
	monolingual	145	10.83	2.92
	Total sample	290	9.84	2.95
Picture Naming	simultaneous bilingual	68	9.49	2.76
	sequential bilingual	77	8.03	2.79
	monolingual	145	10.81	2.73
	Total sample	290	9.76	2.98

*Note.* Language background is defined as the number of acquired languages and the way they were acquired during language development.

**Table 4:** Multivariate analysis of variance (MANOVA 1)—main and interaction effects of language background and parental education on the five WPPSI-IV primary index scores and the FSIQ.

Variables			Multivariate Effects						Univariate Effects				
Fixed Factor		DV	Wilk’s λ	*F*	*df*_1_	/*df*_2_	*p*	η^2^_par_	*F*	*df*_1_	/*df*_2_	*p*	η^2^_par_
1	Language background	VCI	**0.912**	**2.154**	**12**	**/548**	**0.013**	**0.045**	**8.535**	**2**	**/278**	**<0.001**	**0.058**
		VSI							2.534	2	/278	0.081	0.018
		FRI							2.908	2	/278	0.056	0.020
		WMI							1.099	2	/278	0.335	0.008
		PSI							0.478	2	/278	0.621	0.003
		FSIQ							**4.499**	**2**	**/278**	**0.012**	**0.031**
2	Parental education	VCI	**0.882**	**1.952**	**18**	**/773**	**0.010**	**0.041**	**5.817**	**3**	**/278**	**<0.001**	**0.059**
		VSI							2.560	3	/278	0.055	0.027
		FRI							1.126	3	/278	0.339	0.012
		WMI							0.797	3	/278	0.496	0.009
		PSI							2.613	3	/278	0.052	0.027
		FSIQ							**4.260**	**3**	**/278**	**0.006**	**0.044**
	Interaction 1 × 2	VCI	0.840	1.353	36	/1202	0.081	0.029	**2.290**	**6**	**/278**	**0.036**	**0.047**
		VSI							1.528	6	/278	0.169	0.032
		FRI							2.082	6	/278	0.055	0.043
		WMI							**2.507**	**6**	**/278**	**0.022**	**0.051**
		PSI							1.411	6	/278	0.210	0.030
		FSIQ							**2.851**	**6**	**/278**	**0.010**	**0.058**

*Note.* Language background is defined as the number of acquired languages and the way they were acquired during language development (1 = simultaneous bilingual, 2 = sequential bilingual, 3 = monolingual). Parental education is defined as the highest level of education achieved by either one parent or both (1 = no to low educational level, 2 = medium educational level, 3 = high educational level, 4 = highest educational level). DV = dependent variable. VCI = Verbal Comprehension Index, VSI = Visual Spatial Index, FRI = Fluid Reasoning Index, WMI = Working Memory Index, PSI = Processing Speed Index, FSIQ = Full Scale IQ. η^2^_par_ = partial eta squared. Significant multivariate and univariate effects (*p* < 0.050) are marked in bold.

**Table 5:** Post hoc analyses for the main effects of language background and parental education on the WPPSI-IV primary index scores and the FSIQ (MANOVA 1).

Variables				Descriptive Statistics			Test Statistics (Post Hoc)				
DV	Fixed Factor	Factor Level		*N*	*M*	*SD*	Com-parison	Δ*M*	*SE*	*p*	CI (95%)
VCI	Language background	1	bi_sim	68	101.62	13.32	**1 vs. 2**	**6.06**	**2.355**	**0.032**	**0.39; 11.73**
		2	bi_se	77	95.56	14.11	1 vs. 3	−3.09	2.080	0.417	−8.10; 1.92
		3	mono	145	104.70	15.92	**2 vs. 3**	**−9.15**	**1.996**	**<0.001**	**−13.95; −4.34**
	Parental education	1	no/low	68	99.22	17.04	1 vs. 2	2.87	2.321	>0.999	−3.30; 9.04
		2	medium	82	96.35	13.77	1 vs. 3	−4.74	2.637	0.440	−11.75; 2.27
		3	high	50	103.96	12.64	**1 vs. 4**	**−7.49**	**2.274**	**0.007**	**−13.53; −1.45**
		4	highest	90	106.71	14.92	**2 vs. 3**	**−7.61**	**2.540**	**0.018**	**−14.35; −0.86**
							**2 vs. 4**	**−10.36**	**2.161**	**<0.001**	**−16.10; −4.62**
							3 vs. 4	−2.75	2.496	>0.999	−9.38; 3.88
FSIQ	Language background	1	bi_sim	68	103.57	13.48	1 vs. 2	5.52	2.330	0.062	−0.19; 11.03
		2	bi_se	77	98.16	14.08	1 vs. 3	−0.96	2.058	>0.999	−5.91; 4.00
		3	mono	145	104.53	15.25	**2 vs. 3**	**−6.38**	**1.974**	**0.004**	**−11.13; −1.62**
	Parental education	1	no/low	68	99.35	16.99	1 vs. 2	−0.27	2.296	>0.999	−5.83; 6.37
		2	medium	82	99.62	13.14	1 vs. 3	−6.01	2.608	0.132	−12.94; 0.92
		3	high	50	105.36	14.95	**1 vs. 4**	**−6.92**	**2.250**	**0.014**	**−12.90; −0.95**
		4	highest	90	106.28	13.22	2 vs. 3	−5.74	2.512	0.139	−12.41; 0.94
							**2 vs. 4**	**−6.66**	**2.138**	**0.012**	**−12.34; −0.98**
							3 vs. 4	−0.92	2.470	>0.999	−7.48; 5.64

*Note.* DV = dependent variable. VCI = Verbal Comprehension Index, FSIQ = Full Scale IQ. Language background is defined as the number of acquired languages and the way they were acquired during language development (1 bi_sim = simultaneous bilingual, 2 bi_se = sequential bilingual, 3 mono = monolingual). Parental education is defined as the highest level of education achieved by either one parent or both (1 no/low = no to low educational level, 2 medium = medium educational level, 3 high = high educational level, 4 highest = highest educational level). Significant multivariate and univariate effects (*p* < 0.050) are marked in bold.

**Table 6:** Multivariate analysis of variance (MANOVA 2)—main and interaction effects of language background and parental education on two WPPSI-IV ancillary index scores.

Variables			Multivariate Effects						Univariate Effects				
Fixed factor		DV	Wilk’s λ	*F*	*df*_1_	/*df*_2_	*p*	η^2^_par_	*F*	*df*_1_	/*df*_2_	*p*	η^2^_par_
1	Language background	VAI	**0.804**	**15.959**	**4**	**/554**	**<0.001**	**0.103**	**29.473**	**2**	**/278**	**<0.001**	**0.175**
		NVI							2.373	2	/278	0.095	0.017
2	Parental education	VAI	**0.936**	**3.112**	**6**	**/554**	**0.005**	**0.033**	**4.432**	**3**	**/278**	**0.005**	**0.046**
		NVI							2.530	3	/278	0.058	0.029
	Interaction 1 × 2	VAI	**0.923**	**1.883**	**12**	**/554**	**0.034**	**0.039**	0.973	6	/278	0.444	0.021
		NVI							**2.702**	**6**	**/278**	**0.014**	**0.055**

*Note.* Language background is defined as the number of acquired languages and the way they were acquired during language development (1 = simultaneous bilingual, 2 = sequential bilingual, 3 = monolingual). Parental education is defined as the highest level of education achieved by either one parent or both (1 = no to low educational level, 2 = medium educational level, 3 = high educational level, 4 = highest educational level). DV = dependent variable. VAI = Vocabulary Acquisition Index, NVI = Nonverbal Index. η^2^_par_ = partial eta squared. Significant multivariate and univariate effects (*p* < 0.050) are marked in bold.

**Table 7:** Post hoc analyses for the main effects of language background and parental education on the WPPSI-IV ancillary index scores (MANOVA 2).

Variables				Descriptive Statistics			Test Statistics (Post Hoc)				
DV	Fixed Factor	Factor Level		*N*	*M*	*SD*	Comparison	Δ*M*	*SE*	*p*	CI (95%)
VAI	Language background	1	bi_sim	68	97.32	12.61	**1 vs. 2**	**8.04**	**2.167**	**<0.001**	**2.82; 13.26**
		2	bi_se	77	89.29	13.06	**1 vs. 3**	**−7.11**	**1.914**	**<0.001**	**−11.72; −2.50**
		3	mono	145	104.43	13.77	**2 vs. 3**	**−15.15**	**1.836**	**<0.001**	**−19.57; −10.73**
	Parental education	1	no/low	68	96.50	17.09	1 vs. 2	1.21	2.136	>0.999	−4.47; 6.88
		2	medium	82	95.29	13.77	1 vs. 3	−3.30	2.426	>0.999	−9.75; 3.15
		3	high	50	99.80	14.43	**1 vs. 4**	**−6.50**	**2.093**	**0.013**	**−12.06; −0.94**
		4	highest	90	103.00	13.36	2 vs. 3	−4.51	2.337	0.329	−10.72; 1.70
							**2 vs. 4**	**−7.71**	**1.988**	**<0.001**	**−12.99; −2.42**
							3 vs. 4	−3.20	2.297	0.988	−9.30; 2.90

*Note.* DV = dependent variable. VAI = Vocabulary Acquisition Index. Language background is defined as the number of acquired languages and the way they were acquired during language development (1 bi_sim = simultaneous bilingual, 2 bi_se = sequential bilingual, 3 mono = monolingual). Parental education is defined as the highest level of education achieved by either one parent or both (1 no/low = no to low educational level, 2 medium = medium educational level, 3 high = high educational level, 4 highest = highest educational level). Significant differences between factor levels (*p* < 0.050) are marked in bold.

**Table 8:** Multivariate analysis of variance (MANOVA 3)—main and interaction effects of language background and parental education on the 15 WPPSI-IV subtest scaled scores.

Variables			Multivariate Effects						Univariate Effects				
Fixed Factor		DV	Wilk’s λ	*F*	*df*_1_	/*df*_2_	*p*	η^2^_par_	*F*	*df*_1_	/*df*_2_	*p*	η^2^_par_
1	Language background	BD	**0.724**	**3.083**	**30**	**/528**	**<0.001**	**0.149**	1.104	2	/278	0.333	0.008
		IN							**11.121**	**2**	**/278**	**<0.001**	**0.074**
		MR							2.121	2	/278	0.122	0.015
		BS							0.072	2	/278	0.931	0.001
		PM							2.150	2	/278	0.118	0.015
		SI							**3.162**	**2**	**/278**	**0.044**	**0.022**
		PC							2.008	2	/278	0.136	0.014
		CA							1.711	2	/278	0.183	0.012
		ZL							0.345	2	/278	0.709	0.002
		OA							**5.055**	**2**	**/278**	**0.007**	**0.035**
		VC							**10.379**	**2**	**/278**	**<0.001**	**0.069**
		AC							0.244	2	/278	0.783	0.002
		CO							**5.435**	**2**	**/278**	**0.005**	**0.038**
		RV							**20.156**	**2**	**/278**	**<0.001**	**0.127**
		PN							**23.266**	**2**	**/278**	**<0.001**	**0.143**
2	Parental education	BD	**0.737**	**1.885**	**45**	**/785**	**<0.001**	**0.097**	2.213	3	/278	0.087	0.023
		IN							**7.150**	**3**	**/278**	**<0.001**	**0.072**
		MR							0.483	3	/278	0.694	0.005
		BS							**6.206**	**3**	**/278**	**<0.001**	**0.063**
		PM							1.429	3	/278	0.234	0.015
		SI							**5.112**	**3**	**/278**	**0.002**	**0.052**
		PC							1.083	3	/278	0.356	0.012
		CA							0.966	3	/278	0.409	0.010
		ZL							0.554	3	/278	0.646	0.006
		OA							2.192	3	/278	0.089	0.023
		VC							**3.317**	**3**	**/278**	**0.020**	**0.035**
		AC							**3.174**	**3**	**/278**	**0.025**	**0.033**
		CO							**3.200**	**3**	**/278**	**0.024**	**0.033**
		RV							**3.804**	**3**	**/278**	**0.011**	**0.039**
		PN							**3.198**	**3**	**/278**	**0.024**	**0.033**
	Interaction 1 × 2	BD	0.701	1.081	90	/1491	0.289	0.057	1.447	6	/278	0.197	0.030
		IN							1.500	6	/278	0.178	0.031
		MR							1.897	6	/278	0.081	0.039
		BS							1.747	6	/278	0.110	0.039
		PM							1.917	6	/278	0.078	0.040
		SI							**2.154**	**6**	**/278**	**0.048**	**0.044**
		PC							1.595	6	/278	0.149	0.033
		CA							0.812	6	/278	0.561	0.017
		ZL							1.799	6	/278	0.099	0.037
		OA							1.318	6	/278	0.249	0.028
		VC							**2.234**	**6**	**/278**	**0.040**	**0.046**
		AC							0.903	6	/278	0.493	0.019
		CO							0.949	6	/278	0.460	0.020
		RV							1.263	6	/278	0.275	0.027
		PN							0.490	6	/278	0.816	0.010

*Note.* Language background is defined as the number of acquired languages and the way they were acquired during language development (1 = simultaneous bilingual, 2 = sequential bilingual, 3 = monolingual). Parental education is defined as the highest level of education achieved by either one parent or both (1 = no to low educational level, 2 = medium educational level, 3 = high educational level, 4 = highest educational level). BD = Block Design, IN = Information, MR = Matrix Reasoning, BS = Bug Search, PM = Picture Memory, SI = Similarities, PC = Picture Concepts, CA = Cancellation, ZL = Zoo Locations, OA = Object Assembly, VC = Vocabulary, AC = Animal Coding, CO = Comprehension, RV = Receptive Vocabulary, PN = Picture Naming. η^2^_par_ = partial eta squared. Significant multivariate and univariate effects (*p* < 0.050) are marked in bold.

**Table 9:** Post hoc analyses for the main effects of language background and parental education on the WPPSI-IV subtest scaled scores (MANOVA 3).

Variables				Descriptive Statistics			Test Statistics (Post Hoc)				
DV	Fixed Factor	Factor Level		*N*	*M*	*SD*	Comparison	Δ*M*	*SE*	*p*	CI (95%)
IN	Language background	1	bi_sim	68	10.21	2.60	**1 vs. 2**	**1.34**	**0.460**	**0.012**	**0.23; 2.44**
		2	bi_se	77	8.87	2.97	1 vs. 3	−0.64	0.406	0.345	−1.62; 0.34
		3	mono	145	10.85	3.01	**2 vs. 3**	**−1.98**	**0.390**	**<0.001**	**−2.92; −1.04**

	Parental education	1	no/low	68	10.28	3.08	**1 vs. 2**	**1.46**	**0.453**	**0.008**	**0.26; 2.67**
		2	medium	82	8.82	2.76	1 vs. 3	−0.26	0.515	>0.999	−1.63; 1.11
		3	high	50	10.54	2.84	1 vs. 4	−0.84	0.444	0.352	−2.02; 0.34
		4	highest	90	11.12	2.88	**2 vs. 3**	**−1.72**	**0.496**	**0.004**	**−3.04; −0.41**
							**2 vs. 4**	**−2.31**	**0.422**	**<0.001**	**−3.43; −1.18**
							3 vs. 4	−0.58	0.487	>0.999	−1.88; 0.71
BS	Parental education	1	no/low	68	9.09	3.20	**1 vs. 2**	**−1.39**	**0.500**	**0.036**	**−2.72; −0.06**
		2	medium	82	10.48	3.18	**1 vs. 3**	**−2.07**	**0.568**	**0.002**	**−3.58; −0.56**
		3	high	50	11.16	3.14	**1 vs. 4**	**−1.38**	**0.490**	**0.032**	**−2.68; −0.08**
		4	highest	90	10.47	2.81	2 vs. 3	−0.68	0.547	>0.999	−2.14; 0.77
							2 vs. 4	0.01	0.466	>0.999	−1.23; 1.25
							3 vs. 4	0.69	0.538	>0.999	−0.74; 2.12
SI	Language background	1	bi_sim	68	10.47	3.10	1 vs. 2	0.79	0.494	0.325	−0.39; 1.98
		2	bi_se	77	9.68	2.81	1 vs. 3	−0.44	0.436	0.942	−1.49; 0.61
		3	mono	145	10.91	3.20	**2 vs. 3**	**−1.24**	**0.418**	**0.010**	**−2.24; −0.23**

	Parental education	1	no/low	68	9.53	3.41	1 vs. 2	−0.46	0.486	>0.999	−1.75; 0.83
		2	medium	82	9.99	2.92	**1 vs. 3**	**−1.47**	**0.552**	**0.049**	**−2.94; 0.01**
		3	high	50	11.00	2.57	**1 vs. 4**	**−1.83**	**0.477**	**<0.001**	**−3.09; −0.56**
		4	highest	90	11.36	3.07	2 vs. 3	−1.01	0.532	0.349	−2.43; 0.40
							**2 vs. 4**	**−1.37**	**0.453**	**0.017**	**−2.57; −0.16**
							3 vs. 4	−0.36	0.523	>0.999	−1.75; 1.03
OA	Language background	1	bi_sim	68	10.40	2.93	1 vs. 2	0.45	0.478	>0.999	−0.70; 1.60
		2	bi_se	77	9.95	2.87	1 vs. 3	−0.67	0.422	0.339	−1.69; 0.35
		3	mono	145	11.07	2.90	**2 vs. 3**	**−1.12**	**0.405**	**0.018**	**−2.10; −0.14**
VC	Language background	1	bi_sim	68	10.54	2.79	**1 vs. 2**	**1.69**	**0.449**	**<0.001**	**0.61; 2.77**
		2	bi_se	77	8.86	3.01	1 vs. 3	−0.22	0.397	>0.999	−1.18; 0.73
		3	mono	145	10.77	2.62	**2 vs. 3**	**−1.91**	**0.381**	**<0.001**	**−2.82; −0.99**
	Parental education	1	no/low	68	9.60	2.87	1 vs. 2	−0,19	0.443	>0.999	−1.37; 0.99
		2	medium	82	9.79	2.92	**1 vs. 3**	**−1.36**	**0.503**	**0.044**	**−2.69; −0.02**
		3	high	50	10.96	2.73	1 vs. 4	−1.02	0.434	0.117	−2.17; 0.13
		4	highest	90	10.62	2.81	2 vs. 3	−1.17	0.484	0.099	−2.45; 0.12
							2 vs. 4	−0.83	0.412	0.270	−1.92; 0.27
							3 vs. 4	0.34	0.476	>0.999	−0.93; 1.60
AC	Parental education	1	no/low	68	9.76	3.17	1 vs. 2	−0.86	0.494	0.504	−2.17; 0.46
		2	medium	82	10.62	3.03	**1 vs. 3**	**−1.48**	**0.561**	**0.049**	**−2.97; 0.02**
		3	high	50	11.24	2.80	1 vs. 4	−0.85	0.484	0.490	−2.13; 0.44
		4	highest	90	10.61	2.96	2 vs. 3	−0.62	0.541	> 0.999	−2.06; 0.82
							2 vs. 4	0.01	0.460	> 0.999	−1.21; 1.23
							3 vs. 4	0.63	0.532	> 0.999	−0.78; 2.04
CO	Language background	1	bi_sim	68	10.38	2.78	**1 vs. 2**	**1.37**	**0.476**	**0.013**	**0.22; 2.52**
		2	bi_se	77	9.01	2.89	1 vs. 3	0.12	0.421	> 0.999	−0.89; 1.13
		3	mono	145	10.26	2.96	**2 vs. 3**	**−1.25**	**0.404**	**0.007**	**−2.22; −0.28**
	Parental education	1	no/low	68	9.22	2.66	1 vs. 2	−0.39	0.469	> 0.999	−1.64; 0.86
		2	medium	82	9.61	3.00	**1 vs. 3**	**−1.42**	**0.533**	**0.049**	**−2.84; 0.01**
		3	high	50	10.64	3.10	**1 vs. 4**	**−1.23**	**0.460**	**0.046**	**−2.46; −0.01**
		4	highest	90	10.46	2.88	2 vs. 3	−1.03	0.514	0.275	−2.40; 0.33
							2 vs. 4	−0.85	0.437	0.324	−2.01; 0.32
							3 vs. 4	0.18	0.505	> 0.999	−1.16; 1.53
RV	Language background	1	bi_sim	68	9.60	2.57	**1 vs. 2**	**1.40**	**0.445**	**0.006**	**0.32; 2.47**
		2	bi_se	77	8.21	2.53	**1 vs. 3**	**−1.22**	**0.393**	**0.006**	**−2.17; −0.28**
		3	mono	145	10.83	2.92	**2 vs. 3**	**−2.62**	**0.377**	**< 0.001**	**−3.53; −1.71**
	Parental education	1	no/low	68	9.35	3.31	1 vs. 2	0.12	0.439	> 0.999	−1.05; 1.29
		2	medium	82	9.23	2.69	1 vs. 3	−0.59	0.499	> 0.999	−1.91; 0.74
		3	high	50	9.94	2.97	**1 vs. 4**	**−1.37**	**0.430**	**0.010**	**−2.51; −0.23**
		4	highest	90	10.72	2.67	2 vs. 3	−0.71	0.480	0.849	−1.98; 0.57
							**2 vs. 4**	**−1.49**	**0.409**	**0.002**	**−2.58; −0.40**
							3 vs. 4	−0.78	0.472	0.592	−2.04; 0.47
PN	Language background	1	bi_sim	68	9.49	2.76	**1 vs. 2**	**1.46**	**0.455**	**0.004**	**0.36; 2.55**
		2	bi_se	77	8.03	2.79	**1 vs. 3**	**−1.32**	**0.401**	**0.003**	**−2.29; −0.35**
		3	mono	145	10.81	2.73	**2 vs. 3**	**−2.78**	**0.385**	**< 0.001**	**−3.71; −1.85**

	Parental education	1	no/low	68	9.41	3.32	1 vs. 2	0.28	0.448	> 0.999	−0.91; 1.47
		2	medium	82	9.13	2.71	1 vs. 3	−0.65	0.509	> 0.999	−2.00; 0.70
		3	high	50	10.06	2.80	1 vs. 4	−1.01	0.439	0.132	−2.18; 0.16
		4	highest	90	10.42	2.93	2 vs. 3	−0.93	0.490	0.359	−2.23; 0.38
							**2 vs. 4**	**−1.29**	**0.417**	**0.013**	**−2.40; −0.18**
							3 vs. 4	−0.36	0.482	> 0.999	−1.64; 0.92

Note. DV = dependent variable. IN = Information, BS = Bug Search, SI = Similarities, OA = Object Assembly, VC = Vocabulary, AC = Animal Coding, CO = Comprehension, RV = Receptive Vocabulary, PN = Picture Naming. Language background is defined as the number of acquired languages and the way they were acquired during language development (1 bi_sim = simultaneous bilingual, 2 bi_se = sequential bilingual, 3 mono = monolingual). Parental education is defined as the highest level of education achieved by either one parent or both (1 no/low = no to low educational level, 2 medium = medium educational level, 3 high = high educational level, 4 highest = highest educational level). Significant differences between factor levels (*p* < 0.050) are marked in bold.

## Data Availability

Since the research is based on health service research (Versorgungsforschung) the data are not publicly available.
